# Self‐Supervised App‐Based Speech Training for Children With Speech Sound Disorder—A Single‐Case Experimental Design Study

**DOI:** 10.1111/1460-6984.70163

**Published:** 2025-12-01

**Authors:** Sofia Strömbergsson, Ella Edlund, Magdalena Pettersson, Nhan Phan, Mikko Kurimo

**Affiliations:** ^1^ Division of Speech and Language Pathology, Department of Clinical Science, Intervention and Technology (CLINTEC) Karolinska Institutet Stockholm Sweden; ^2^ Department of Neurology, Division of Speech and Language Pathology Danderyd Hospital Stockholm Sweden; ^3^ Department of Information and Communications Engineering Aalto University Espoo Finland

**Keywords:** intervention, speech sound disorder, speech technology

## Abstract

**Background:**

For children with speech sound disorder (SSD), speech intervention often involves a considerable amount of home‐training, to achieve high‐enough training frequency to promote speech change. A digital speech‐training app has been developed that could serve as a cost‐effective means of providing accessible intervention to children with SSD.

**Aims:**

To evaluate whether self‐supervised home‐training with the app *Pop2TalkNordic* can expedite more target‐like speech for children with SSD and to explore children's experiences of using the app.

**Methods and Procedures:**

Four 4–6‐year‐old Swedish children with SSD participated in a single‐case experimental design study, with a multiple‐baseline across‐subjects design. The children's production of target error patterns was monitored during baseline and intervention phases, for trained and untrained stimulus words. Three weeks of self‐supervised training with the app, with an aspired frequency of 5 days a week, in 15‐min training sessions, served as the intervention. The children's app usage was tracked, and their production of target word stimuli was recorded via the app.

**Outcomes and Results:**

None of the children reached more target‐like production of targeted consonants as a result of the intervention. For two participants, slight improvement was observed on trained, but not untrained, word stimuli. In terms of user experiences, the children varied from liking the game a lot and finding it easy, to not liking the game much at all and finding it difficult.

**Conclusions and Implications:**

In its current form, and when delivered as a self‐supervised training‐activity over three weeks, training with *Pop2TalkNordic* is not sufficient to expedite more target‐like speech in children with SSD. More parental engagement in the children's training with the app, and changes in game design (e.g., highlighting phonological contrast and allowing playback of multiple exemplars of target word items), are suggested routes to achieve better outcomes.

**WHAT THIS PAPER ADDS:**

*What is already known on this subject*

- To achieve high‐enough intervention dose, home‐training is often an important part of intervention for speech sound disorder (SSD). This is not always easy for families to attain, however, and digital speech training games may offer an attractive alternative or complement.

*What this paper adds to existing knowledge*

- By tracking four children's usage and speech production over three weeks of playing a speech training game at home, the paper shows that the current version of the game and the implemented intervention delivery are not sufficient to promote more accurate production of targeted speech sounds.

*What are the potential or actual clinical implications of this work?*

- Before recommending the speech training game for children with SSD, changes are recommended both with regard to the design of the game itself (e.g., refining the feedback provided in the game) and to the intervention delivery (e.g., increasing parental involvement).

## 1 Introduction

Speech sound disorder (SSD) is one of the most common paediatric conditions at speech‐language pathology clinics (Wren et al. 2018), affecting around 8%–9% of preschool‐aged children (Eadie et al. 2015; Harding et al. 2024; Shriberg et al. 1999). Children are often referred during preschool years, when concerns are raised concerning their speech acquisition. Most often, the difficulties with speech are not associated with a known condition (e.g., a cleft palate or a hearing impairment). This group—SSD with unknown origin—can be further subdivided, primarily with regard to surface display and whether the difficulties are motor‐based (e.g., in the case of Childhood Apraxia of Speech), or not (Stringer et al. 2023). Most often, there are no indications of motor difficulties; this subtype is often referred to as phonological SSD (Baker et al. 2022; Stringer et al. 2023; Sugden et al. 2018).

In phonological SSD, speech is typically characterised by the occurrence of predictable speech error patterns (Dodd 2014). A distinction is made between developmental and atypical error patterns, where developmental patterns are common early in speech acquisition, but may persist in children with SSD at an age when they are no longer expected (Dodd 2014). Two developmental error patterns that are common in many languages are *stopping* and *velar fronting*. Stopping occurs when fricative consonants are produced as plosives, for example, when an expected /s/ or /ʃ/ is produced as [t], or a /v/ is produced as [b] (Brosseau‐Lapré and Rvachew 2020; Strömbergsson et al. 2021). Velar fronting is observed when velar consonants are produced with a more anterior, typically alveolar/dental, place of articulation, for example, when an expected /k/ is produced as [t], or /ŋ/ is produced as [n] (Brosseau‐Lapré and Rvachew 2020; Strömbergsson et al. 2021). For children with SSD, when such systematic error patterns persist over time, speech‐language intervention is often recommended (Baker and McLeod 2011; Sugden et al. 2016).

For phonological SSD, intervention typically has a cognitive‐linguistic focus, engaging the child in tasks highlighting phonological contrasts they are currently not expressing (Baker and McLeod 2004; Wren et al. 2018). For the child, such tasks often involve both listening and producing targeted sounds, at a word level (Baker and McLeod 2004). For example, a therapeutic task tailored for velar fronting, where a child has difficulties producing the phonemic contrast between, for example, /t/ and /k/, may be to, while being presented with pictures illustrating the minimal word pair ‘tea’ and ‘key’, listen to a model speaker producing one of these words, and identify which of the pictures represents the spoken word (Baker 2021). This perceptual identification task could be followed by a production task, where the child is asked to produce one of the two words and to evaluate whether a listener identifies the intended word in line with that intention (Baker 2021). A critical component of phonological intervention is that the assumed reorganisation of the child's phonological system that intervention aims to achieve will result in system‐wide changes in the child's speech (Brosseau‐Lapré and Rvachew 2020). A child exhibiting stopping, for example, who during intervention grasps the notion of ‘fricativeness’ and starts to produce more target‐like /f/, can be expected to soon also be producing more target‐like /s/ and /ʃ/. An important ingredient of intervention delivery is high‐enough dose, involving the child producing target sounds many times during a training session, and high‐enough dose frequency, that is, involving regular scheduling of training sessions (Sugden et al. 2018). Based on their systematic review of phonological intervention, Sugden and colleagues (2018) outline recommendations that treatment should be individual, occur 2–3 times per week, each session lasting for 30–60 min, and including at least 50–100 ‘production efforts’ per treatment session (where ‘production effort’ indicates each time the child is auditorily exposed to, or produces, the targeted sound(s) during a treatment session). Typically, intervention involves a considerable amount of home‐training to achieve this (Sugden et al. 2016).

Indeed, home‐based, parent‐delivered training can be a viable way to achieve the intervention intensity needed to promote speech change in children with SSD (Leafe et al. 2025; Tosh et al. 2017). Leafe et al. (2025) present several factors that may contribute to successful parent‐implemented intervention, such as parent‐training and tailoring training to fit with other family commitments. One potential benefit of parental involvement in speech intervention, as well as a key to increased training intensity, is the opportunity to integrate practice into everyday life activities (Bowen and Cupples 2004; Sugden et al. 2016; Leafe et al. 2025). Despite many benefits of parent‐implemented intervention, however, there are also barriers. For example, parents may find instructions on how to complete practice activities unclear and experience challenges in finding time for practice (Sugden et al. 2019; Doherty et al. 2025). Digital speech‐training tools may provide a route to overcoming barriers to intervention (Leafe et al. 2025). However, although a multitude of digital tools have been developed for children with SSD, the quality control is often insufficient (Furlong et al. 2018), and few have been tested with clinical populations (Wren et al. 2021).

During more recent years, an app‐based speech‐training game has been developed and evaluated for use in Australia: *SayBananas!* (or, in its beta version: *Apraxia World*). This app involves tasks for the child user to both listen and produce selected target words, and is provided with feedback on target word accuracy, as a correct/incorrect decision (Hair et al. 2021; McLeod et al. 2023). In the first evaluation of the app, Hair et al. (2021) studied 10 children (one with a motor‐based SSD, and nine with idiopathic SSD) and their usage of the app over a period of 4 plus 4 weeks (with an intervening 2‐week break). The researchers observed improved target sound accuracy in the children and high levels of engagement throughout the extended intervention. In their subsequent evaluation of intervention for 45 children with (unspecified) SSD over a 4‐week self‐supervised intervention period, McLeod et al. (2023) also observed improved speech accuracy, although not quite as pronounced as after the longer intervention. Another example of a speech‐training app that has been trialled with a clinical population is the *staRt* (*Speech Therapist's App for /r/ Treatment)* app (Peterson et al. 2022), developed specifically for the treatment of residual /r/ errors. In their single‐case experimental design study, Peterson et al. (2022) explored treatment effects of a clinician‐led telepractice intervention using *staRt*, for four children across 16 training sessions. Here, too, the participating children increased their speech target accuracy during intervention. Notably, with regards to Wren and colleagues’ classification of intervention approaches (Wren et al. 2018), both *SayBananas!* and *staRt* can be categorised primarily as production‐oriented, with a basis in principles of motor learning (Maas et al. 2008). For example, they both contain elements of high intensity (i.e., many production efforts) and randomisation of stimuli (Hair et al. 2021; Peterson et al. 2022). In the case of *staRt*, the grounding in motor learning principles is even stronger, as it incorporates knowledge of performance feedback (via visual‐acoustic biofeedback), includes not only words but also non‐lexical CV syllable target items, and entails an elaborate scheme for when and how to increase task difficulty (Peterson et al. 2022). *SayBananas!*, on the other hand, allows an integrated cognitive‐linguistic focus by allowing the selection of target word items that constitute minimal pairs (McLeod et al. 2023). Although the focus of intervention, as well as the format of intervention delivery, varied between these studies, they serve as illustrating examples that speech production accuracy in children with SSD can indeed be expedited in interventions based on speech‐training apps.

In early‐stage evaluation research, such as when evaluating new interventions where effects are uncertain, *single‐case experimental design (SCED)* is recommended as a study design (Krasny‐Pacini 2023). Further, the design is appreciated for having high clinical relevance as a method of exploring changes within individual patients (Maggu et al. 2021). In studies with this design, outcome measures are prospectively assessed from a small number of participants, at frequent intervals, before and during treatment (Krasny‐Pacini 2023). Measurements taken during the phase preceding treatment constitute the baseline, to which measurements taken during and after treatment are compared. The experimental control of the design, for example, by using randomised treatment conditions or multiple baselines, ensures internal validity, allowing treatment effects to be distinguished from maturation effects (Krasny‐Pacini 2023). With a stable baseline, a pronounced change during the treatment phase is interpreted as a consequence of the intervention. In this sense, each participant serves as their own control (Krasny‐Pacini 2023). Within the research area of intervention for children with SSD, the SCED is a well‐established study design (e.g., Gomez et al. 2024; Maas et al. 2019; Peterson et al. 2022; Preston et al. 2018).

**FIGURE 1:**
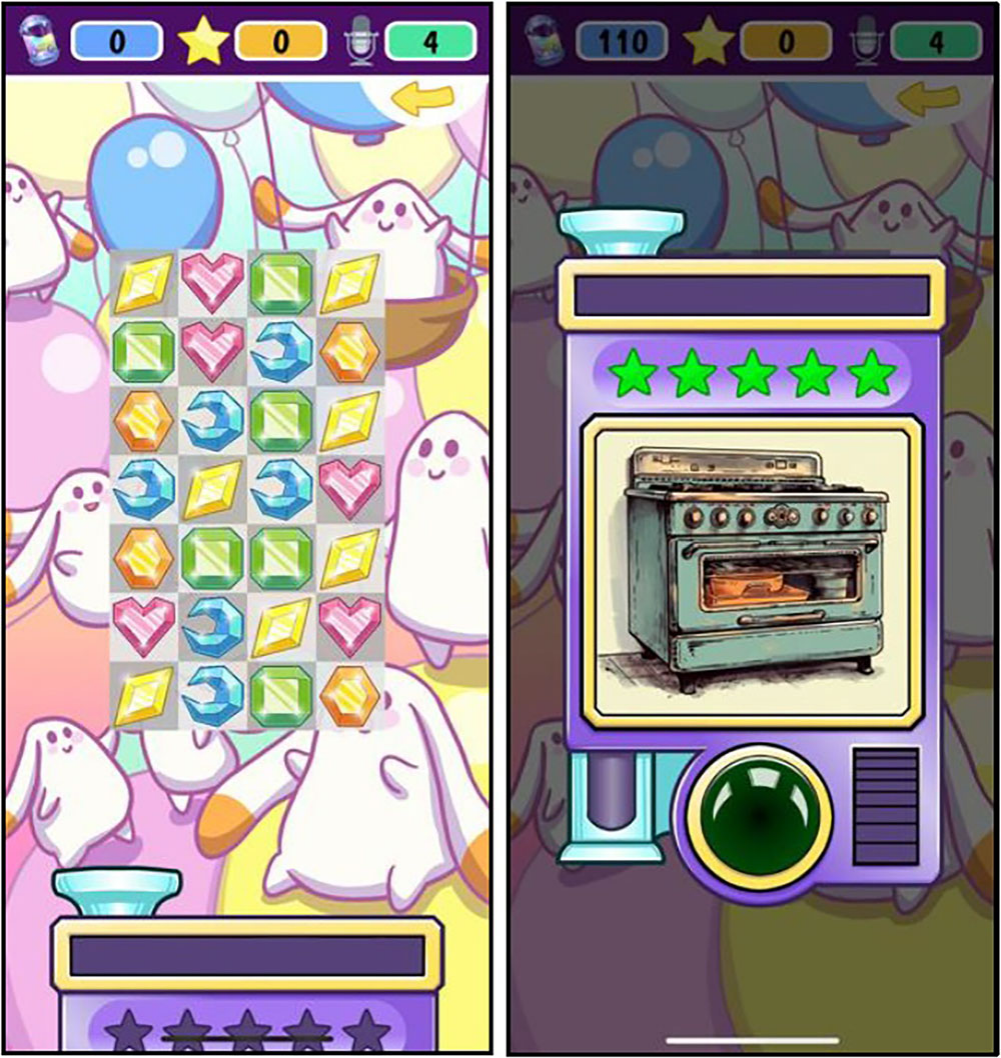
Two screenshots of the game in *Pop2TalkNordic*, illustrating the game. The screenshot to the left illustrates the playing interface during a gaming level. Here, the user's actions elicit the playback of an audio file (in this case, a recording of the word 'ugn' /ɵŋn/, Eng. *oven*). The screenshot to the right illustrates the game after having completed the level, when the user has recorded their own production of the word 'ugn', and receives a 5‐star rating as feedback.

The present study is the first to evaluate the use of the speech‐training app *Pop2TalkNordic* (Getman et al. 2023), and whether it can expedite the acquisition of targeted consonant sounds for Swedish children with SSD. *Pop2TalkNordic* is an app designed for speech‐training, embedded in a Candy Crush‐like game, see Figure 1. While playing, the user is auditorily exposed to playback of pre‐selected word stimuli and subsequently requested to produce the word stimuli. Automatic speech recognition is integrated in the app, enabling the automatic assessment of the user's production of target words. The automatic assessment is presented as feedback to the user, as a rating of one to five stars, see Figure 1 (for a demonstration of the app, see https://www.youtube.com/watch?v=S8L0lIpWyKg or https://aalto.cloud.panopto.eu/Panopto/Pages/Viewer.aspx?id=73a36336‐d6eb‐4821‐a26c‐b3270013b7c1.

Although a previous version of the app has been used in the context of Finnish‐speaking children learning English as a second language (Junttila 2022; Ylinen et al. 2021), the app has not been evaluated as an intervention for children with SSD before. From an SSD intervention perspective, *Pop2TalkNordic* contains several components that have been found beneficial in SSD intervention. For example, repeated playback of target words reflects a perceptual component, modelling the acoustic‐phonetic target word form. The elicitation of the user's child's own production reflects an element of articulatory practice. By providing the child with feedback on their pronunciation of target words, the app also provides external knowledge of results feedback. Provided that the feedback is accurate, this may constitute a valuable active ingredient in an intervention. But even if the app, in theory, may have features that are known to be beneficial in intervention for children with SSD, this remains to be empirically evaluated.

### 1.1 Aim and Research Questions

The aim of this study was to examine whether home‐based training with the app *Pop2TalkNordic* can expedite more target‐like speech for Swedish children with SSD who exhibit systematic speech error patterns. Using a single‐case experimental design, the following research question was investigated:

*Does production accuracy of targeted speech sounds improve, in trained and untrained stimulus words, during baseline and intervention phases?*

An observation of higher accuracy of targeted speech sounds during the intervention phase compared to the baseline phase would suggest that target accuracy improves as a consequence of intervention. A more pronounced improvement in trained stimulus words compared to untrained stimulus words would strengthen the interpretation that improvements are indeed driven by the intervention.

An additional aim was to explore children's experiences of using the app, through the following research question:

*Is there an association between intervention outcome and the children's experience of using the app?*

An association was expected, such that children showing more signs of progression would also have more positive experiences of using the app, compared to children for whom progression was smaller or did not occur at all. In addition, intervention dose and frequency were expected to be associated both with intervention outcome and user experience, or, simply put: children who enjoy playing, can be expected to spend more time playing, and therefore, show greater improvements.

Lastly, a tertiary aim was to investigate the accuracy of the 1–5‐star feedback provided to the children while playing the game, by exploring:

*How well do ratings provided in the game correspond to perceptual assessment of the children's efforts at producing target words?*

If high correspondence is found, the feedback can be regarded as a potentially active ingredient in intervention. If no or little correspondence is found, however, the feedback would constitute random noise that may or may not function as general encouragement to the children while playing.

## 2 Method

The study is a part of the *TEFLON* project (PI: author MK; local PI: author SS), approved by the Swedish Ethical Review Authority (registration number 2022‐05299‐01). In line with this, all participating children and their caregivers provided their informed consent to participation.

### 2.1 Participants

The inclusion criteria for participation were an age of 4–8 years and an SSD diagnosis with speech assessment confirming either stopping or velar fronting as a systematic speech pattern. The criterion of exhibiting either stopping or velar fronting was motivated by the fact that the game was available in two versions: one targeting stopping and one targeting velar fronting. Ongoing speech therapy was set as an exclusion criterion to avoid interference in the tracking of change during intervention with the app. Information about the study was distributed via speech‐language pathologists (SLPs) in the Stockholm region, and caregivers interested in participation contacted the project assistant to volunteer. Twelve families volunteered. Eight children were excluded either because they did not meet the inclusion criteria or because it was not possible to schedule their participation in line with the study protocol. In the end, four children participated: Adam, Benjamin, Carl and Doris (pseudonyms). By caregiver report, none of the children had any conditions such as autism, ADHD or hearing impairment. All children attended preschool, and all used Swedish as their only language. Information concerning previous SLP treatment and its contents was sparse; all participants had ongoing or previous SLP contact, but none of them was enrolled in SLP treatment during their participation in the study. Table 1 presents descriptive information of the four participants, including results from speech assessment at study intake. As shown in the table, all children passed the oral‐motor screening without remark, and all exhibited developmental speech error patterns.

**TABLE 1:** Descriptive information of participants at study intake, concerning age, and speech assessment with regards to oral‐motor function (NOT‐S; Bakke et al. (2007) speech error patterns and perception of target phonological contrast (SP‐PT; Locke 1980).

Child	Age (y;m)	Oral motor function	Speech error pattern(s)	Speech perception	PCC	PWC [age reference M; SD]^a^
Adam	4;6	Without remark	Stopping of voiceless fricatives (in medial and final position), consonant cluster reduction	Difficulties perceiving the /s/‐/t/ contrast	73%	43% [83%; 19]
Benjamin	4;4	Without remark	Fronting of velar sounds, in all word positions (except for /k/ in medial and final position), /r/ weakening, consonant cluster reduction	Perceiving the /t/‐/k/ contrast	58%	23% [90%; 96]
Carl	4;11	Without remark	Fronting of /k/ and /ŋ/ in final position, and of /g/ in initial position, /r/ weakening, /l/ ‐> [j], consonant cluster reduction	N/A^b^	59%	30% [83%; 19]
Doris	6;2	Without remark	Fronting of velar consonants (albeit occasional target accurate initial /k/ and /g/), stopping of fricatives (primarily in initial position), /r/ weakening, consonant cluster reduction	Perceiving the /t/‐/k/ contrast, but difficulties perceiving the /s/‐/t/ contrast^a^	44%	8% [98%; 0]

^a^Reference values from Lundeborg Hammarström (2019). Quantitative measures of speech production are presented with respect to percentage of consonants correct (PCC; Shriberg and Kwiatkowski 1982) and percentage of words correct (PWC), as based on LINUS 2.0 (Lundeborg Hammarström 2019). Error patterns targeted in intervention are underlined in the description of speech error patterns.

^b^ For Carl, assessment was interrupted and could not be completed. Doris occasionally responded by pointing in‐between yes and no; these responses were tallied as incorrect.

### 2.2 Materials

Information concerning demography and general development (see above) was collected from the children's caregivers via a digital questionnaire. The intention was also to collect caregiver report concerning the children's functional intelligibility via a digital version of the Intelligibility in Context Scale (ICS; McLeod et al. 2012), but due to a technical error, this form was never distributed.

The children were assessed by a project assistant (author MP; a certified SLP), to confirm the SSD diagnosis, as well as to provide information concerning their speech profiles. Oral motor function was assessed with NOT‐S (Bakke et al. 2007) to exclude speech and/or oral motor difficulties. Articulation/expressive phonology was assessed with LINUS 2.0 (Lundeborg Hammarström 2019), to confirm and detail the children's difficulties with producing fricatives and/or velar consonants. The short version of LINUS 2.0 was used, comprising 40 target words (12 monosyllabic, 21 disyllabic and 7 three‐syllable words), with target fricatives and velar consonants represented 24 and 19 times, respectively. Based on this material, speech error patterns were identified (see Table 1). Based on the same material, the percentage of consonants correct (PCC; Shriberg and Kwiatkowski 1982) was calculated as a quantification of speech accuracy (see Table 1). The participating children's performance on LINUS 2.0 was also compared to norm references (Lundeborg Hammarström 2019) with respect to the percentage of words correct (PWC), see Table 1. To assess the children's perception of the phonological contrast they had difficulties producing, the Speech Perception‐Production Task (SP‐PT, Locke 1980) was used. For Adam, the task was tailored to their stopping of fricatives, such that a picture of a *sun* (Swedish: “sol”, /su:l/) was shown, and recorded audio prompts of the format “Is this X?” were alternated, where X was either /su:l/ (i.e., correct), /tu:l/ (i.e., incorrect, in line with their expected substitution for the target /s/), or /fu:l/ (i.e., incorrect, but not expected in their production). The task for the children was to respond yes or no to the recorded question. For Benjamin and Carl, the task was tailored to their fronting of velar consonants, with a picture of a *comb* (Swedish: “kam”, /kam/) being presented, and with the task for the children to whether the picture depicted /kam/ (correct), /tam/ (incorrect; expected substitution), or /pam/ (incorrect; unexpected substitution). Doris completed the task tailored to both her stopping and velar fronting, as per above. All assessments were audio‐recorded.

To provide data for the outcome measure, that is, the speech accuracy of target speech sounds, word lists of 24 words were tailored to the children's target error patterns. These word lists were used as *probes* during the baseline and intervention phase of the study (see below). The word lists contained words with the target sounds (/k, g, ŋ/ for velar fronting, and /s, ɕ, ʂ, ɧ/ for stopping) in initial, medial and final position. Half of the words (i.e., 12 words) occurred as training items in the intervention, and the other half did not. The complete word lists are available as Table S1 (for stopping) and Table S2 (for velar fronting).

The intervention comprised training with the app *Pop2TalkNordic* (Getman et al. 2023), see Figure 1. With the app, the user plays a game made up of multiple levels. While playing a level, a recording of a word is played as a response to the user's actions. After completing the level, the recorded word is played again one final time, while a picture depicting the word is presented. Then, the user is requested to produce the word, listen to their recorded pronunciation of the word, and get feedback from the app, presented with regards to a scale from one to five stars. At the final step, the user can reproduce the word as many times as they want, including listening to the recorded sample and getting feedback. The automatic assessment was based on acoustic models trained on global ratings of whole word accuracy, conducted by SLP listeners; for technical details, see Getman et al. 2023). After this, the user continues to the next level, with a new word as target. The *Pop2TalkNordic* app is publicly available and can be downloaded via Google Play or the Apple App Store (via an unlisted link). Login credentials can be provided upon request, strictly for demonstration purposes.

The participating children were assigned one of the two different game versions, tailored to their specific speech targets. For Adam, the game targeted stopping, and for Benjamin, Carl and Doris, the game targeted velar fronting. For Adam, Benjamin, and Carl, this target was developmentally motivated, whereas for Doris—who exhibited both stopping and velar fronting—velar fronting was prioritised as a target based on its more systematic presence, compared to stopping. In designing the sets of target words for each of the two game versions (i.e., the stopping and fronting versions), efforts were made to (a) balance the distribution of target sounds in initial, medial, and final positions, (b) achieve variation in terms of word shape complexity (number of syllables, lexical stress). Apart from that, the selected words should be easily depicted and assumed to be familiar to children from 4 years of age. (This was not critical, however, as the target words were always presented in the game with an adult voice modelling the production of the word.) The final sets of target words contained 86 and 93 items, for stopping and velar fronting, respectively. The complete set of words included in the different game versions is listed in Table S3 (for stopping) and Table S4 (for velar fronting). The game was designed with an increasing level of difficulty with the user's progression through levels, starting with less complex (monosyllabic, homorganic) target words at lower levels, and progressing to more complex words on higher levels.

At the end of the intervention period, the families were requested to fill out a questionnaire concerning their experiences of using the app. The questionnaire consisted of seven questions with fixed response alternatives (‘not at all’, ‘a little,’ ‘pretty much,’ and ‘a lot’), and two open‐ended questions. The questionnaire targeted the children's thoughts about the game, for example, if they enjoyed practicing with the game, and whether they felt that it was easier to produce difficult sounds after having practiced with the game. The questions were directed towards the children, but caregivers were asked to assist them in responding. The caregivers could also add their own comments. The questionnaire is included as Appendix S5.

### 2.3 Study Design

**FIGURE 2:**
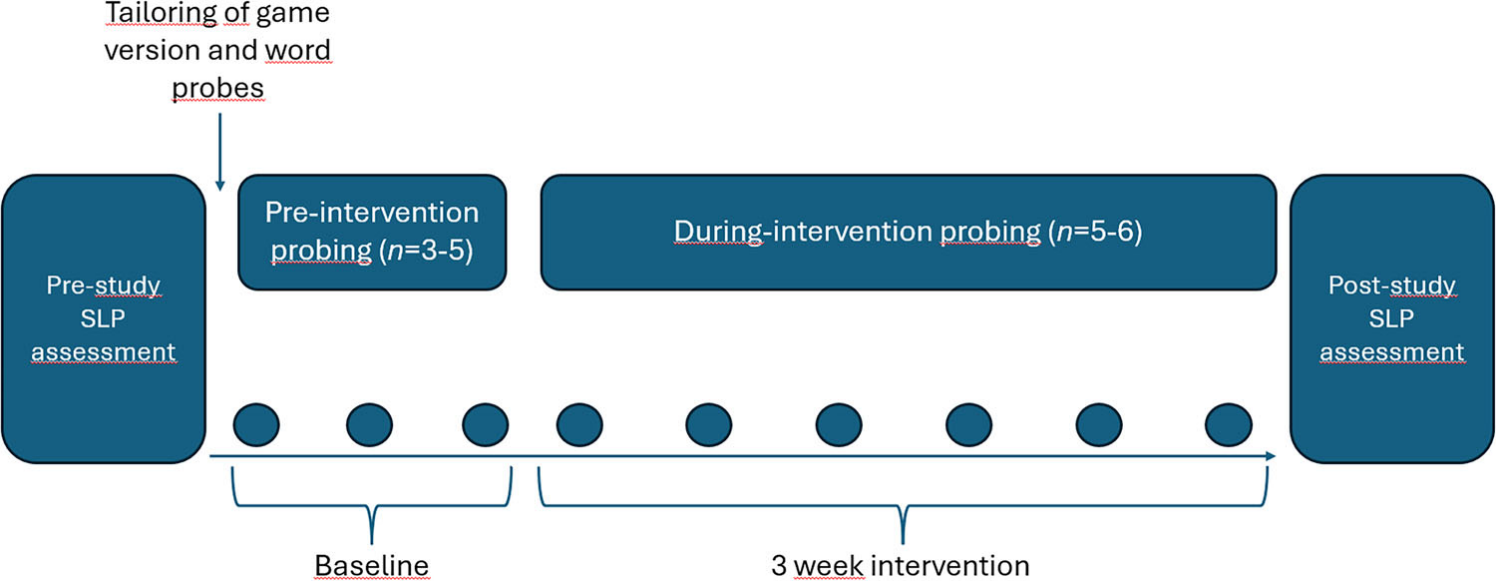
A stylised presentation of the data collection procedure, following the single‐case experimental design, with a multiple‐baseline across‐subjects design. Note that the number of baseline probes was randomised as one of the numbers 3, 4 and 5 and therefore varied across participants, as indicated in parentheses. The number of intervention probes also varied across participants (between 5 and 6), for practical reasons related to the scheduling to complete the probe measures.

The study employed a single‐case experimental design (SCED), following a multiple‐baseline across‐subjects design. The accuracy of target consonants was probed regularly across baseline and intervention phases, see Figure 2. The multiple‐baseline across‐subjects design involved random assignment of participants to baseline phases of different lengths, following the design in Preston et al. (2018). These restrictions serve to strengthen the internal validity, that is, to allow conclusions regarding whether potential progress is the result of the intervention or not (Krasny‐Pacini 2023). With the number of baseline probes being randomised as one of the numbers 3, 4 and 5, Adam and Carl were assigned three baseline probes, and Benjamin and Doris were assigned five baseline probes. Broad assessment of speech production was conducted at study intake and after intervention by means of LINUS 2.0 (Lundeborg Hammarström 2019).

### 2.4 Procedures

The SLP assessment followed clinical routine and was conducted in the children's homes by a project assistant (author MP, a certified SLP). Following assessment, probe word lists were tailored to reflect the participating children's speech error patterns, and the children were randomly allocated to three, four or five baseline probe sessions. Baseline probe sessions were scheduled twice a week with the project assistant on Zoom. During the probe sessions, the child was presented with pictures representing the target probe words and asked to name the pictures while being recorded.

After the final baseline probe session, the child and their caregivers were given access to the speech‐training app for downloading to a device of their preference. Before commencing the intervention phase, the children and their caregivers were instructed to practice with the app 15 min a day, 5 days a week, for 3 weeks. They were encouraged to stick to the 15 min as far as possible, but if their child could not maintain motivation throughout the full 15 min, they were instructed to encourage, but not to force their child to continue. If they could not fit in training for 1 day, they were encouraged to try again the next day. All tasks with the speech game were conducted at home, by the child with the support of their caregivers. The caregivers were instructed to make sure that the child could hear the prompts in the game, with or without headphones. Audio recordings of the children's efforts at producing the words in the speech‐training application were collected and uploaded to a secure server during training, and information concerning their gaming activity was logged. The activity logs contained information regarding what target words were used (played back and elicited) during each session.

The project assistant monitored and supported the families during the intervention phase by continuing the regular probe sessions twice a week. The procedure for these probe sessions was the same as during baseline. For practical reasons related to the scheduling of probe sessions during intervention, the number of probe sessions varied across the participants, with Adam and Benjamin completing five sessions, and Carl and Doris completing six. The time between probe sessions varied between 2 and 9 days for the participants. The last probe session was conducted at the end of the intervention phase. In that same session, conducted in the families’ homes, a broad assessment of articulation/expressive phonology with LINUS 2.0 (Lundeborg Hammarström 2019) was repeated.

### 2.5 Analysis

Target accuracy was perceptually evaluated and documented as a binary measure: 1 if the target consonant in a recorded word was perceived as matching the target pronunciation, and 0 if it did not. Production of non‐targeted sounds was disregarded. Some target words contained multiple instances of target sounds, for example, ‘ryggsäck’ /ɹˈʏɡsˌɛk/ (Eng. *backpack*) for velar consonant targets; in such cases, all target consonants (i.e., both /g/ and /k/) had to match the target for target accuracy to be documented as 1. As an outcome measure, the target accuracy of all words in a probe session was compiled as the proportion of words in the session where consonant target accuracy was 1; this measure will henceforth be referred to as target accuracy percentage (TA%).

For the tracking of potential progress towards more accurate production of target consonants, line diagrams were generated, with TA% as the dependent variable. A result where TA% is stable at or close to 0 during baseline, and markedly increases towards 100% during the intervention phase, would constitute strong support that the intervention was successful. Observed progress (i.e., increased TA%) on *trained* probe items would indicate a positive intervention effect. Observed progress on *untrained* probe items would indicate that the intervention has generalised to untrained items. As a complement to the visual analysis, trend and potential overlap between baseline and intervention phases were analysed by means of Kendall's Tau and Non Overlap of All Pairs (NAP; Manolov and Tanious 2024), respectively. Kendall's Tau assesses trend within a phase (−1: strong decreasing trend, +1: strong increasing trend, 0: no trend). NAP, on the other hand, assesses potential overlap between pairs of baseline and intervention measurements to yield an estimate of intervention effect (NAP > 90%: strong effect, 70%–89%: moderate effect, 50%–69%: weak or questionable effect, <50%: possible reverse effect).

In addition to the tracking of (potential) progression on probe items, the children's speech was also monitored during their training with the app. For these intervention word items, target accuracy was assessed, and TA% calculated following the same protocol as for the probe items. Intervention items were included in analysis as a supplementary measure to track potential progress at a more detailed level than solely via probe measures. (For this analysis, only items evaluated as efforts at producing the target word—that is, not laughter or mere noise—were included.) A result where no difference is seen between baseline and intervention probe items, but where a positive trend during intervention is observed on intervention items, would suggest that some progression has occurred (although not necessarily as a consequence of the intervention). Apart from evaluating the recordings collected during training as the basis of potential progress during intervention, the collected recordings were also tallied as an estimation of the number of ‘production efforts’ during intervention. Information from activity logs was also used as the basis for an estimation of number of auditory exposures to target words. For each level, the user was passively exposed to a target word when playing (usually between 4 and 6 times per level). The target word was also played back at the end of a level, when eliciting the user's own production of the target word. In addition, the user could—as many times as they wished—actively select a ‘retry’ option, to hear the target word again, before producing it again. Consequently, the number of auditory exposures to a target word during each level was estimated as: *4* (as the minimum number of playbacks during playing the level) + *1* + *number of active retries*. The number of exposures for all levels played during a training session was summed into a total number of auditory exposures to target words within that session.

To provide a supplementary measure on a more general level, general speech production was assessed at study intake and after intervention, based on the LINUS 2.0 material. Speech production accuracy on these assessments was quantified with regard to the Percentage of Consonants Correct—Revised (Shriberg et al. 1997; Shriberg and Kwiatkowski 1982), and descriptively documented as per clinical protocol. This would reveal whether potential improvement has generalised to production of non‐targeted sounds.

To provide insight into what feedback the participating children received from the game during intervention, the 1–5‐star ratings presented as feedback on their production of target words were summarised per rating category, and related to the whole‐word based measure of proportion of phonemes correct (Shriberg et al. 1997), and to the perceptually assessed target accuracy (0 or 1). Again, only utterances that were evaluated as efforts at producing the target word were included in analysis.

#### 2.5.1 Reliability

For all perceptual analyses, all word items were randomised (within participant) to ensure that assessors were blinded with regard to the timing of recording. Three evaluators were involved in the perceptual analysis of speech data: author EE (an SLP student at the time, in her final semester of the study programme), author MP, and author SS (both certified SLPs). Author MP perceptually evaluated the accuracy of target consonants in all probe items. For calculation of inter‐rater reliability, author EE perceptually evaluated 30% of this dataset (randomly selected across all participants and probe sessions) following the same procedure. Inter‐rater reliability between the two assessors was estimated by point‐by‐point agreement. Of the 249 recordings that were evaluated by both assessors, 223 were in agreement, resulting in a point‐by‐point agreement of 89.5% (Cohen's kappa = 0.69, reflecting substantial agreement; Landis and Koch 1977). Unfortunately, it was not possible for MP to redo the assessment at a later date; therefore, intra‐rater reliability could not be calculated.

For the intervention items, author EE perceptually evaluated accuracy of target consonants in the full dataset. For estimation of intra‐rater reliability, EE re‐evaluated 30% of the intervention items (randomly selected across participants and intervention sessions) after 2 weeks. Of the 104 items that were evaluated twice, 94 were in agreement, representing a point‐by‐point agreement of 90.4% (Cohen's kappa = 0.74, reflecting substantial agreement; Landis and Koch 1977). For inter‐rater reliability, author SS perceptually evaluated the same 30% of the intervention items. Here, 82 items were in agreement, representing a point‐by‐point agreement of 78.8% (Cohen's kappa = 0.42, reflecting moderate agreement; Landis and Koch 1977).

## 3 Results

### 3.1 Training and outcomes per participant

In the following, intervention outcomes are presented for one participant at a time, together with descriptive data, to enable interpretation of intervention outcomes for each individual child. To aid the interpretation of results, Table 2 presents intervention delivery characteristics in terms of dose frequency, dose and cumulative intervention intensity.

**TABLE 2:** Overview of dose frequency (average number of training sessions per week), dose (average number of trials—Production efforts and/or auditory exposures—Per session), and cumulative intervention intensity, for the four participants.

		Dose			Cumulative intervention intensity		
Child	Dose freq.	Production efforts	Auditory exposures	Total Prod + Aud	Production	Auditory exposure	Total Prod + Aud
Adam	4.7	9.5	43.2	52.7	114	518	632
Benjamin	4.8	14.0	40.9	54.9	154	450	604
Carl	7.0	11.5	37.7	49.2	173	565	738
Doris	4.0	7.2	32.2	39.3	86	386	472

#### 3.1.1 Adam

**FIGURE 3:**
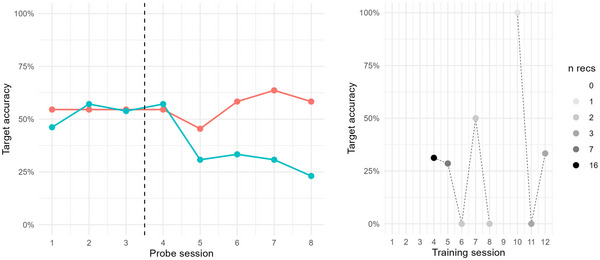
Left: Target accuracy percentage (TA%) for Adam in probe items during baseline and intervention phase, across trained (red) and untrained (blue) probe items. Right: Target accuracy percentage (TA%) for Adam in intervention items during the intervention phase. The number of evaluated recordings per intervention session (*n* recs) is indicated by grey scale colouring.

Adam was assigned three baseline probes; these were collected over the course of 9 days. During this baseline phase target accuracy percentage (TA%) was stable at around 50%, see Figure 3. Intervention through speech training with the app was delivered over 18 days, across 12 sessions, corresponding to a dose frequency of 4.7 sessions/week (see Table 2). According to activity logs, Adam practiced with the app over a total of 86 min, with an average of 7.1 min per training session (min = 2 min, max = 16 min). During this time, he was requested to produce target words 114 times, corresponding to a dose of 9.5 production efforts/session. As for auditory exposure, Adam was exposed to target words during intervention 518 times, corresponding to a dose of 43.2 auditory exposures/session. As Figure 3 shows, the stability of TA% during baseline did not remain after intervention was introduced. Instead, for trained probe items, TA% increased somewhat (confirmed by Kendall's Tau = 0.4, and NAP = 70%, moderate effect, showing that 70% of intervention data points were on a higher level relative to baseline). For untrained probe items, however, TA% decreased (Kendall's Tau = −0.7, NAP = 17%, possible reverse effect). Taken together, these results provide evidence of slight improvement on trained items, but no positive effect of the intervention on Adam's production of fricatives in untrained items.

Closer examination of recordings collected during training was compromised by data scarcity. Across all 12 training sessions, only 50 recordings were made from the app, and of these, 36 were evaluated as efforts at producing the target word and were audible enough to be perceptually assessed. As seen in Figure 3, the one training session where TA% was higher than at any of the probe sessions was the 10th session; however, this measure is based on only one datapoint. Hence, no positive trend could be discerned from the recordings collected via the app during intervention.

Broad assessment of speech production at the end of intervention showed no clear improvement; PCC at 73% at study intake was now 75%. Fricatives were still affected, but slight improvement was observed; for example, /s/ in final position was now produced as [θ] rather than as [t], and /f/ in medial and final position, which was target accurate only occasionally at study intake, was now consistently produced as [f]. Consonant cluster reduction remained at the same level.

In terms of user experiences, Adam was positive towards hearing his own voice in the game and towards being rewarded with stars. Adam found the game quite easy and felt that he was “a little” better at producing the target sounds after having played with the app. When asked if he had suggestions for how the game could be improved, Adam requested more levels. According to his caregivers, Adam reached the final level in the game quite quickly, which seemed to have weakened his motivation to continue training.

#### 3.1.2 Benjamin

**FIGURE 4:**
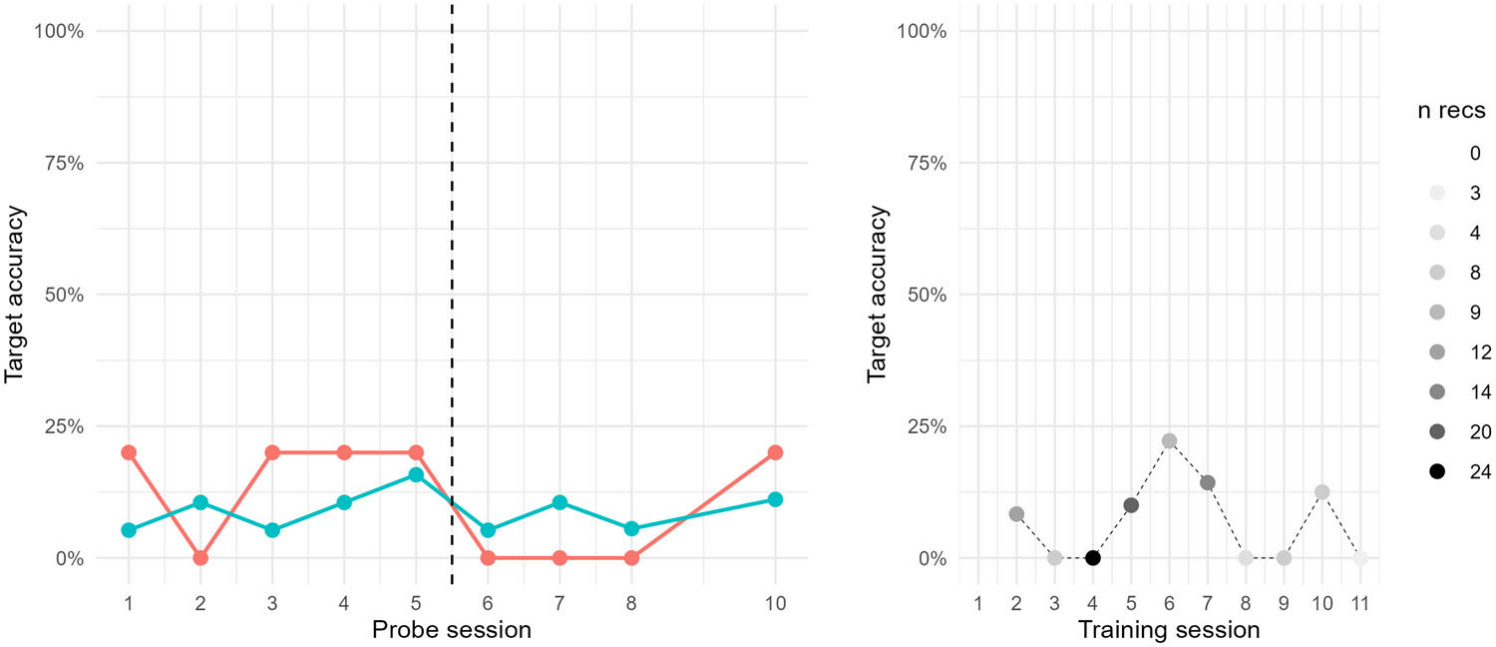
Left: Target accuracy percentage (TA%) for Benjamin in probe items during baseline and intervention phase, across trained (red) and untrained (blue) probe items. Right: Target accuracy percentage (TA%) for Benjamin in intervention items during the intervention phase. The number of evaluated recordings per intervention session (n recs) is indicated by grey scale colouring.

Benjamin was assigned five baseline probes, collected over 16 days. During the baseline phase, target accuracy percentage (TA%) fluctuated somewhat but remained at 20% or lower, see Figure 4. Intervention with the app was delivered over 16 days, across 11 sessions, corresponding to a dose frequency of 4.8 sessions/week. According to activity logs, Benjamin practiced with the app over a total of 96 min, with an average of 8.7 min per training session (min = 3 min, max = 17 min). During this time, he was requested to produce target words 154 times, corresponding to a dose of 14 production efforts/session. Regarding auditory exposure, Benjamin was exposed to target words during intervention 450 times, corresponding to a dose of 40.9 auditory exposures/session. As Figure 4 shows, there was no improvement in TA% during the intervention phase, neither for trained nor for untrained probe items. If anything, the statistical analysis indicates decreased accuracy on trained items (Kendall's Tau = −0.6, NAP = 23%, possible reverse effect), whereas for untrained items, no statistically measurable change could be seen (Kendall's Tau = 0, and NAP = 50%, weak effect). Hence, the results did not show any positive effects of intervention on Benjamin's production of velar sounds.

Across the 11 training sessions, 120 of 142 recordings were evaluated as efforts at producing the target word, and evaluated with respect to TA%. As shown in Figure 4, no progression with respect to TA% in app recordings could be tracked during intervention.

Broad assessment of speech production at the end of intervention showed a slight increase in PCC, from 58% at study intake to 61%. With regards to the targeted velars, initial /g/ was now accurate, whereas /k/—which was occasionally produced correctly at study intake—was now instead consistently produced as [t]. Consonant cluster remained, whereas occasional target‐accurate production of /r/ was observed.

In terms of user experiences, Benjamin found the game fun but difficult. He was positive towards hearing his own voice in the game and towards being rewarded with stars. Benjamin did not experience any improvement in his production of the target sounds, and he did not want to practice any more with the game. As for improvements of the game, Benjamin suggested ‘a little nicer and glitterier, and with a little bit more banana and candy in it’.

#### 3.1.3 Carl

**FIGURE 5:**
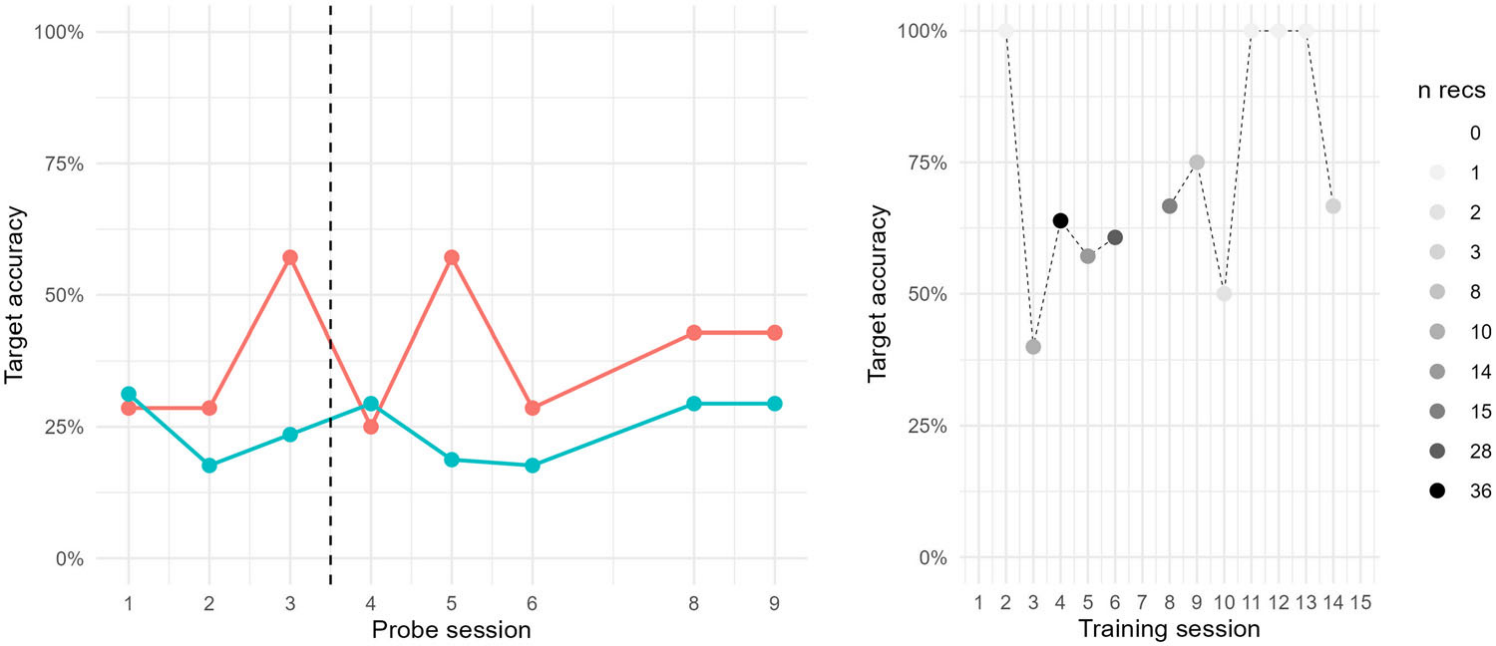
Left: Target accuracy percentage (TA%) for Carl in probe items during baseline and intervention phase, across trained (red) and untrained (blue) probe items. Right: Target accuracy percentage (TA%) for Carl in intervention items during intervention phase. The number of evaluated recordings per intervention session (*n* recs) is indicated by grey scale colouring.

Carl was assigned three baseline probes, collected over 11 days. During this baseline phase, target accuracy percentage (TA%) was unstable, particularly for items that were planned as targets during intervention, see Figure 5. Intervention was delivered over 15 days, across 15 sessions, corresponding to a dose frequency of 7 sessions/week. According to activity logs, Carl practiced with the app over a total of 126 min, with an average of 9 min per training session (min = 0.5 min, max = 23 min). During this time, he was requested to produce target words 173 times, corresponding to a dose of 11.5 production efforts/session. As for auditory exposure, Carl was exposed to target words during intervention 565 times, corresponding to a dose of 37.7 auditory exposures/session. As seen in Figure 5, there is no clear sign of an increase in TA% during the intervention phase, although one might note that trained probe items are generally produced with higher TA% than untrained items. In addition, TA% for trained items at 43% at the last two probe sessions is also higher than the TA% at 29% on the first two probe sessions. Statistically, however, no signs of an intervention effect could be seen, neither for trained items (Kendall's Tau = 0, NAP = 50%), nor for untrained items (Kendall's Tau = 0, NAP = 50%). Hence, there is no suggestion of any positive effect of the intervention on Carl's production of velar consonants.

Across the 15 training sessions, 110 of 142 recordings were evaluated as efforts at producing the target word and evaluated with respect to TA%. As seen in Figure 5, TA% for several training sessions are based on a few data points, compromising the discerning of a trend across sessions. However, it can be noted that for three training sessions, TA% is based on at least 10 data points, with levels at around 60%, that is, higher than for the probe sessions. This may be seen as a sign of subtle improvement during intervention.

Broad assessment of speech production at the end of intervention showed a quite substantial increase with regard to PCC; from 59% at study intake to 68%. As for the targeted velars, however, fronting remained at the same level. Increased accuracy was instead observed in production of /l/ and consonant clusters, although /r/ weakening remained throughout.

In terms of user experiences, Carl expressed that he liked the game a lot and that he found the game very easy. He liked being rewarded with stars, and he liked to hear recordings of his own speech “a little bit”. His caregivers were also positive towards the app, presenting the user with recordings of their own speech. Carl experienced that he had become better at producing the target sounds, and he was mildly positive towards practicing more at producing the sounds, and with the game. As for improvements of the game, Carl and his caregivers suggested that more variation in the game play boards would have made it more motivational.

#### 3.1.4 Doris

**FIGURE 6:**
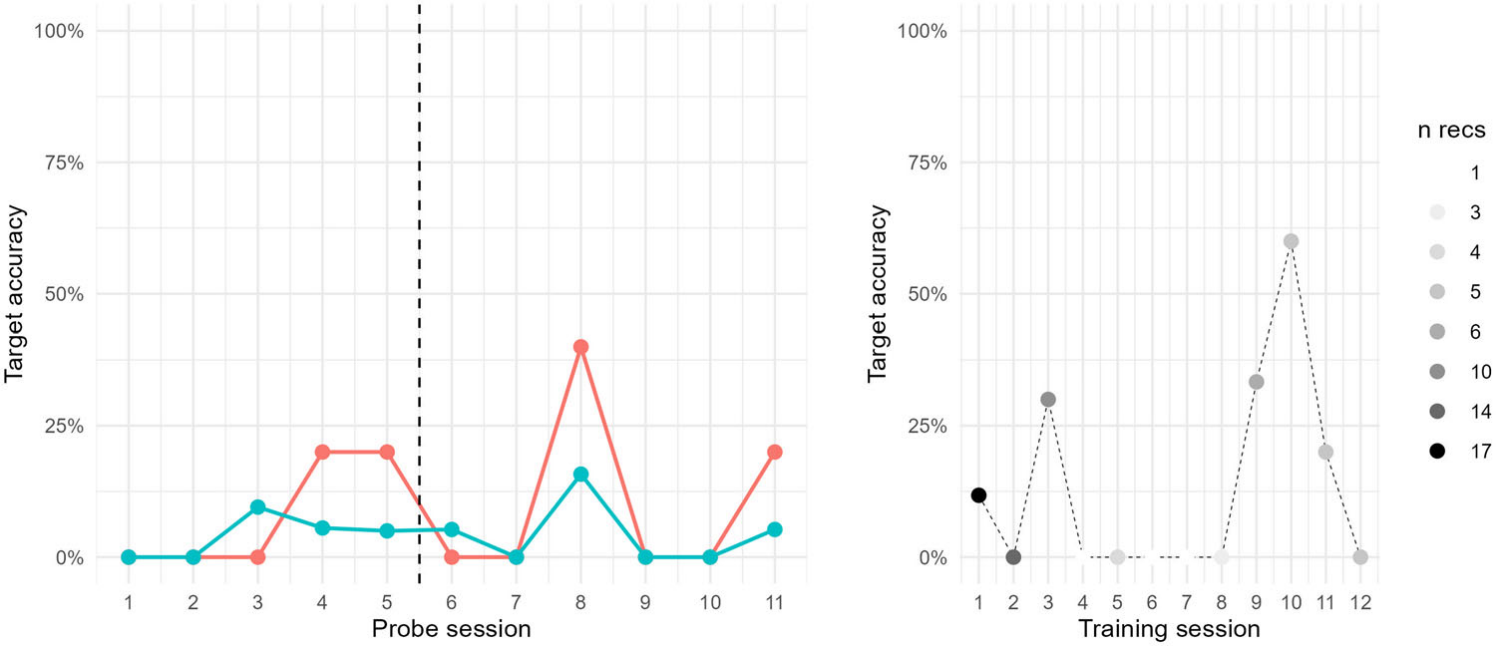
Left: Target accuracy percentage (TA%) for Doris in probe items during baseline and intervention phase, across trained (red) and untrained (blue) probe items. Right: Target accuracy percentage (TA%) for Doris in intervention items during intervention phase. The number of evaluated recordings per intervention session (*n* recs) is indicated by grey scale colouring.

Doris was assigned five baseline probes, collected over 14 days. During this baseline phase, target accuracy percentage (TA%) was quite stable below or around 10%, except for (soon to be) trained items that reached 20% on two sessions, see Figure 6. Intervention was delivered over 21 days, across 12 sessions, corresponding to a dose frequency of 4 sessions/week. According to activity logs, Doris practiced with the app over a total of 83 min, with an average of 7.5 min per training session (min = 1.7 min, max = 14 min). During this time, she was requested to produce target words 86 times, corresponding to a dose of 7.2 production efforts/session. Regarding auditory exposure, Doris was exposed to target words during intervention 386 times, corresponding to a dose of 32.2 auditory exposures/session. As seen in Figure 6, there is no clear sign of increase in TA% during the intervention phase, although some fluctuation can be noted. Statistically, no signs of improvement could be seen, neither for trained items (Kendall's Tau = 0, NAP = 50%), nor for untrained items (Kendall's Tau = −0.1, NAP = 47%). In sum, there is no suggestion of any positive effect of the intervention on Doris’ production of velar consonants.

Across the 12 training sessions, 72 of 86 recordings were evaluated as efforts at producing the target word, and evaluated with respect to TA%. Just like the probe measurements during the intervention phase, TA% of app recordings during the intervention phase is fluctuating, with a maximum at 60% at the 10th training session, but then returning to 0% two sessions later. These fluctuations make it impossible to discern certain signs of progression in app recordings during intervention.

Broad assessment of speech production at the end of intervention showed no change; PCC at 45% at study intake was now 45%. The targeted velars were consistently fronted, as compared to occasional accurate production of /k/ and /g/ in initial position at study intake. Stopping remained on some fricatives, although for /v/ and /s/, production was now accurate in all positions. /r/ weakening and consonant cluster reduction remained.

In response to the user experience questionnaire, Doris expressed that she found the game difficult and that she did not like it. She was positive, however, to being rewarded with stars, and towards hearing her own recorded speech in the game. Doris did not think she had become better at producing the target sounds, and she did not want to play the game again, although she was slightly positive towards continued speech training in general. As for suggested improvements, Doris and her family suggested the possibility of selecting one target sound at a time, for practicing this over a couple of weeks, with increasing levels of difficulty. In addition, they suggested clearer indication for when the user was expected to produce their utterance for recording, as Doris had found this difficult.

### 3.2 Feedback Accuracy

**FIGURE 7:**
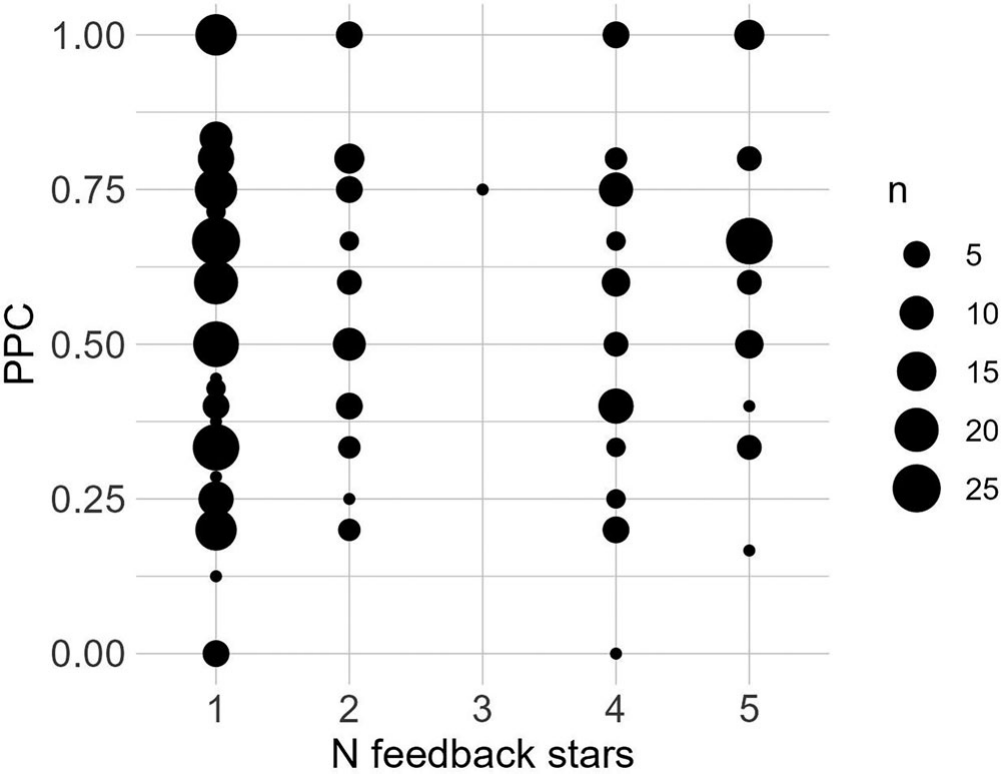
Correspondence between the ASR‐based number of stars (1–5) provided as feedback in the game, and the perceptually evaluated whole‐word accuracy, as measured by the percentage of phonemes correct (PPC; Shriberg et al. 1997).

Regarding the accuracy of feedback provided in the game, Figure 7 illustrates the association between the ASR‐based 1–5‐star feedback ratings and the perceptually evaluated whole‐word accuracy. As seen, there is little correspondence, suggesting that the feedback provided in the game does not reflect the overall speech accuracy in the children's efforts at producing target words. Further, as Table 3 illustrates, the distribution of 1–5‐star ratings is similar across utterances perceptually evaluated as target accurate (i.e., where TA = 1), compared to those evaluated as not being target accurate (i.e., where TA = 0). In other words, the game feedback also does not correspond to whether the targeted sounds were produced accurately or not. Taken together, these results illustrate a low degree of feedback accuracy. The distribution of star ratings for each participant is presented in Table S6.

**TABLE 3:** The distribution of ASR‐based star ratings (1–5) for utterances perceptually evaluated as target accurate (i.e., where TA = 1), and utterances evaluated as target inaccurate (i.e., where TA = 0).

	% Ratings per rating category					
TA	1	2	3	4	5	*n* ratings
1	56%	19%	N/A	13%	12%	104
0	57%	10%	0%	16%	16%	234

## 4 Discussion

The primary aim of this study was to evaluate whether children with systematic speech error patterns could achieve more accurate production of target consonants as a consequence of training with *Pop2TalkNordic*, and in addition, if intervention outcome could be linked to the children's experience of using the app. Overall, no clear intervention effect could be seen. For two of the children, Adam and Carl, slight improvement was observed for target consonants in trained word items compared to target consonants in untrained word items. In terms of user experiences, the children varied from liking the game a lot and finding it easy, to not liking the game much at all and finding it difficult. Of the four children, Adam and Carl were the only ones who experienced that they had become “a little bit better” at producing the target sounds after intervention. Further, feedback accuracy was explored, showing that the 1–5‐star ratings provided as feedback in the game to the children's efforts at producing target sounds did not reflect perceptual assessments.

The results that intervention with *Pop2TalkNordic* was not successful for the participating children runs counter to the positive results reported in previous studies of app‐based speech intervention for children with SSD (Hair et al. 2021; McLeod et al. 2023; Peterson et al. 2022). There are several possible explanations for this that can be related to features of the intervention delivery, the participants’ profiles, and the digital tool.

In terms of intervention delivery, the current study employed a shorter and less supervised form of intervention as compared to previous investigations. Compared to the 4 plus 4 weeks in Hair et al. (2021), the 4‐week intervention in McLeod et al. (2023), and the 5–8‐week intervention in Peterson et al. (2022), the duration of three weeks in the present study was considerably shorter. Regarding dose, the observed dose of 7 to 14 production efforts per session in the present study is remarkably lower than recommended levels of 50–100 ‘production efforts’ per session, as suggested by Sugden and colleagues (2018) in their review. Note, however, that ‘production effort’ in Sugden et al. (2018) also includes auditory exposure. Indeed, if the estimated auditory exposure is also included as ‘production efforts’, the observed dose for Adam and Benjamin (52.7 and 54.9, respectively) reaches the recommended range, whereas the observed dose for Doris (49.2) is just short of reaching it, and for Carl (39.3) does not reach it. Compared to the observed dose of 76 production efforts in Hair et al. (2021), and 200 in Peterson et al. (2022), it is markedly lower, whereas it is more similar to the 45 observed in McLeod et al. (2023). It should be noted, however, that there is a critical difference between these speech‐training tools, where *Pop2TalkNordic* offers more auditory exposure to target words than opportunities to production compared to both *staRt* and *SayBananas!*. Regarding dose frequency, on the other hand, the observed frequency ranging between four and seven sessions per week in the present study, is comparable to the frequency of four sessions per week in Hair et al. (2021) and McLeod et al. (2023), and higher than the frequency of 2–3 sessions per week in Peterson et al. (2022). It is possible that intervention with *Pop2TalkNordic* with a longer duration and higher dose would increase the likelihood of improved speech accuracy. However, at this early stage, we were reluctant to engage children and families to potentially ineffective interventions for longer time than necessary. A path to increasing dose could be to encourage the use of a timer to motivate the children to engage with the game for the recommended 15 min per session. Based on the usage logs, it was apparent that the average time spent playing per session was considerably shorter (7–9 min). Even though better support for reaching the recommended session duration does not allow control of the number of ‘production efforts’ per session, it would increase the likelihood that dose reaches and surpasses the recommended minimum level of 50 per session (Sugden et al. 2018). As regards supervision, the intervention described in Hair et al. (2021) and McLeod et al. (2012) required parents to participate with their child during their practice with the app. And in Peterson et al. (2022), all training sessions were conducted with guidance from the child's SLP via video call. The intervention in the current study was less supervised. Apart from regular digital calls with the project assistant to follow up the intervention and to record probes, the families were left unsupervised. Although caregivers were instructed to ensure good auditory conditions (e.g., limiting surrounding noise, and using headphones) for their child during practicing with the game, they were not instructed to engage with their child's practice (as in, e.g., McLeod et al. 2023 and Hair et al. 2021). It is possible that more parental engagement would help the children sustain attention and motivation during practice. A short pre‐practice before each training session, to remind the children of the target sounds and—if needed—to provide feedback of their production of target sounds (as in McLeod et al. 2023) could also be considered. Although McLeod et al. (2023) noted parents requesting the possibility of their children to practice more independently with the game, more parental engagement should at least be considered as a modification to the intervention delivery.

In terms of participant profiles, Furlong et al. (2017) identified factors associated with improvement of speech accuracy with digital intervention, such as being younger (4–5‐year‐olds as compared to 6–7‐year‐olds), being female, being stimulable for target sounds and exhibiting accurate phoneme perception before intervention. The participants in the present study exhibited variation across these factors; for example, Doris was the only girl, but also the oldest of the participating children. The observation that she did not improve target accuracy might potentially be related to her speech difficulties being more persistent than in the younger participants. In addition, the lack of improvement could also be associated with the fact that she did not like the game and had, by far, the lowest cumulative intervention intensity of the participants. The observation that Carl liked the game a lot, and had the highest cumulative intervention intensity, could, conversely, be associated with his subtle signs of progression on trained word items. Still, it can be noted that Benjamin, who also enjoyed the game, and had similar cumulative intervention intensity as Adam, showed no signs of improvement. For Adam and Carl, who both showed improved accuracy on trained word items (although for Carl, the improvement was subtle), it is difficult to identify features that distinguish them from the other two participants. Instead, Carl stands out as the one whose general speech accuracy increased. However, as he only showed subtle increase in target accuracy on consonants targeted in intervention, and only on trained word items, the improvement cannot be attributed to the intervention but could be a consequence of maturation. As for speech perception, an observation could potentially explain the somewhat more favourable outcomes for Adam and Carl, as compared to Benjamin and Doris. Benjamin and Doris both passed the assessment without remark, whereas Adam had difficulties with phonemic perception of his targeted error pattern, and Carl's speech perception assessment was incomplete. Considering that accurate phoneme perception before intervention has been identified as a favourable factor for digital intervention (Furlong et al. 2017), this may appear counter intuitive. But again, given that *Pop2TalkNordic* involves more focus on auditory exposure than on speech production, it may be that Adam and Carl benefitted more from training with the app, than Benjamin and Doris, who did not have difficulties with phoneme perception. This interpretation resonates the findings in Wolfe et al. (2003), where an advantage in speech production accuracy was observed for children who demonstrated perceptual difficulties prior to intervention, compared to peers who did not, after SSD intervention with a perceptual focus (SAILS; see Rvachew and Brosseau‐Lapré 2021).

Regarding the digital tool, there are several aspects of *Pop2TalkNordic* that should be considered in the interpretation of results. First, it should be acknowledged that although *Pop2TalkNordic* does entail components that are recommended in intervention for SSD, it was not designed with this population in mind. It is possible that a different game design, more closely aligning with evidence‐based intervention foci (see Wren et al. 2018), would have resulted in better outcomes. For closer alignment with a cognitive‐linguistic intervention focus, exercises highlighting the phonological contrast could be integrated into the game, for example by focusing on meaningful minimal contrast (Baker and McLeod 2004). And considering the game's focus on auditory exposure to target words, an auditory‐perceptual intervention focus could be strengthened by including not only one prototypical model pronunciation per target word, but multiple exemplars, to reflect the acoustic‐phonetic variability comprised in the phonemic category in focus (see e.g., Rvachew and Brosseau‐Lapré 2021). Also, increased opportunities for individual tailoring—for example, regarding the selection of target words, and the order in which they are presented—should be considered in future versions of the game. In the current version (including the two pre‐sets for ‘stopping’ and ‘fronting’, respectively), the target word lists and their order of presentation are set. Another route of development is the automatic assessment that is integrated into the app. Indeed, the lack of correspondence between the ASR‐based 1–5‐star ratings provided as feedback in the game to clinical measures of speech accuracy is noteworthy. Again, this falls back to the fact that the game—with its 1–5 rating scale and its speech assessment mechanics—was not designed with phonological SSD in mind. As elaborated in (Strömbergsson et al. 2024), the use of the 1–5 rating scale in the context of SSD can be questioned; both SLP clinicians and non‐expert adults have difficulties using the scale in their ratings of children's disordered speech. In the context of phonological SSD, clinicians typically disregard non‐targeted sounds and focus their attention to the targeted sounds when providing feedback in therapy (Hall et al. 1998). The speech assessment mechanics in *Pop2TalkNordic*, however, relies on holistic word comprehension, rather than on explicit modelling of phonemes or other subword units. As such, the feedback provided in the game is not explicitly tailored to the children's production of targeted consonants. That being said, it is worth noting that none of the child users commented on the feedback provided by the app; instead, they all reported that they enjoyed receiving stars. So even though the feedback ratings were not reflective of clinical feedback, it is possible that the star ratings did play a role in keeping the children engaged with the game.

### 4.1 Limitations and Future Directions

Unfortunately, there were some technical issues that made the implementation of the study divert from original plans. First, there was a failure in the digital distribution of the ICS form to the participating children's caregivers. Unfortunately, this failure was not discovered until the end of the study. Still, although this left out an important aspect of the description of the participating children's communicative profile, it does not invalidate the evaluation of intervention. Second, there were technical problems with recording via the app, which resulted in a loss of documented efforts at producing target words. Together with the fact that not all recorded utterances could be evaluated as efforts at producing the target words, either because of noise in the recordings, or because the children were producing other sounds (e.g., laughter, or utterances unrelated to the target words), this resulted in missing data from practice sessions. As for the evaluation of intervention, however, this data loss did not affect the main outcome measure. For future development, one could consider the suggestion by Hair et al. (2021), to develop automatic detection of low recording quality, to prompt the user to adjust microphone placement and/or change recording environment.

It should also be noted that the pre‐intervention assessment was, in some ways, incomplete. Ideally, more effort should have been spent on ensuring reliable assessment of speech perception, as this is a factor that may play a decisive role in intervention outcome (Wolfe et al. 2003). Unfortunately, the present study leaves room for uncertainty in this regard. In addition, the assessment of stimulability should have followed a standard protocol (see e.g., Glaspey and Stoel‐Gammon 2005). The incidental nature of the assessment leaves room for uncertainty also in this regard.

In addition, the outcome measure—target accurate (0/1)—could be questioned. Indeed, it is based on a coarse‐grained level of analysis—with a binary decision of whether target consonants were produced accurately or not. Considering the fine‐phonetic detail that can be observed in children's consonants during acquisition (Strömbergsson et al. 2015; Wikse Barrow et al. 2022), it is possible that a more fine‐grained evaluation of target‐likeness would have been sensitive to more subtle signs of progression in the children's speech production.

The limited insights into user experiences, both from the children's and caregivers’ perspectives, should also be acknowledged; indeed, the user evaluation questionnaire filled out after the intervention does not provide much detail. Although *Pop2TalkNordic* user experiences have been investigated before (Uther et al. 2018), this was done with a population of 8 to 12‐year‐olds who used the app for second language learning. As such, previous findings may not generalise to the participants in the present study. We hypothesise that limited interest in the game, compounded by relatively strict automated feedback, produced a poor user experience for some children and a perception of high task difficulty. For instance, three participants (Benjamin, Carl and Doris) had sessions with repetitions of the same word without an improved star rating; in one of Benjamin's sessions, the same word was attempted 16 times, with attention waning and late attempts showing increase in pitch and laughter. Regardless of the accuracy of the ASR‐based feedback, repeated 1‐star outcomes on successive attempts are likely to be frustrating. Two design changes could plausibly mitigate this. First, assistive progression—auto‐advancing to a new item after a small number of unsuccessful attempts—is consistent with dynamic difficulty adjustment principles (Zohaib 2018). Second, adding effort‐based rewards alongside accuracy rewards to maintain engagement (Mueller and Dweck 1998). For example, small ‘effort stars’ or badges for retries even without accuracy gains (Sailer et al. 2017). Evaluating such design elements was beyond our scope, but they are concrete targets for future user experience studies. For future investigations, we also recommend that more information about user experiences be gathered, for example, via interviews and drawings (see e.g., McLeod et al. 2023), and involving both SLP and human‐computer interaction perspectives. Until then, investigations of parent‐implemented digital intervention for SSD (e.g., Leafe et al. 2025), and clinician perspectives on app design for SLP intervention (e.g., Du et al. 2023), provide rich sources of information that could guide the design and implementation of successful intervention.

Finally, for future versions of the game, other speech assessment mechanisms could be considered. A promising method that could be beneficial in the context of phonological SSD is mispronunciation detection and diagnosis (MDD), which is a focus in computer‐assisted pronunciation training research. If that line of direction is embarked upon, however, it comes with the challenge of obtaining the necessary annotated data to implement MDD (Lounis et al. 2024), especially for a small language like Swedish (Langlais et al. 1998). In addition to updating the speech assessment mechanism, a more coarse‐grained scale—more similar to the three‐grade scale in *staRt* (Peterson et al. 2022), or the binary correct/incorrect decision in *SayBananas!* (Hair et al. 2021; McLeod et al. 2023)—could be considered in future versions of the app. Until the feedback from the app is fully reliable, one could consider involving the caregivers in complementing or substituting feedback from the app with their feedback (as in Hair et al. 2021; McLeod et al. 2023).

### 4.2 Conclusion

In its current form, and when delivered as a home‐based unsupervised intervention across three weeks, engagement with the speech training game *Pop2TalkNordic* cannot be expected to promote more target‐like speech for children with phonological SSD. Before trials with new participants are done, modifications are recommended both with regard to the service delivery and to the speech training app itself. Regarding service delivery, more parental engagement and efforts to achieve a higher dose are suggested. As for development of the game, re‐designing for better alignment with evidence‐based intervention for children with SSD should be considered (e.g., by highlighting phonological contrast), together with re‐consideration of the 1–5‐star feedback scale, and efforts to achieve higher feedback accuracy (e.g., by using a different speech assessment mechanism).

## Funding

This research has been funded by NordForsk (project no. 103893).

## Conflicts of Interest

The authors report no conflicts of interest.

## Supporting information




**Supplementary *Table S1***: Word probes (*n* = 24) selected for participants with intervention targeting stopping. Note that a single word can contain multiple target consonants, i.e. fricatives. (Tone 2/grave accent is indicated with a superscript ^2^.)
**Supplementary *Table S2***: Word probes (*n* = 24) selected for participants with intervention targeting velar fronting. (Note that a single word can contain multiple target consonants, i.e. velar consonants.)

## Data Availability

Data, R scripts and supplementary data can be found at https://osf.io/xw8rd/.
