# Supplementary material for: Self‐Supervised App‐Based Speech Training for Children With Speech Sound Disorder—A Single‐Case Experimental Design Study
Source: Int J Lang Commun Disord. 2025 Dec 1;61(1):e70163. doi: 10.1111/1460-6984.70163 (PMC12670065; doi:10.1111/1460-6984.70163)
Supplement: Supplementary file 1 — Supplementary Table S1 : Word probes (n = 24) selected for participants with intervention targeting stopping. Note that a single word can contain multiple target consonants, i.e. fricatives. (Tone 2/grave accent is indicated with a superscript 2.) Supplementary Table S2 : Word probes (n = 24) selected for participants with intervention targeting velar fronting. (Note that a single word can contain multiple target consonants, i.e. velar consonants.) [file JLCD-61-0-s001.docx]

*Table S1.* Word probes (*n* = 24) selected for participants with intervention targeting stopping. Note that a single word can contain multiple target consonants, i.e. fricatives. (Tone 2/grave accent is indicated with a superscript ^2^.)

| **Word** | ***English translation*** | **Target form** | **Target sound word position(s)** | **Trained (0=no, 1=yes)** |
| --- | --- | --- | --- | --- |
| ryggsäck | *backpack* | /ɹ^2^ʏɡsˌɛk/ | MED | 1 |
| kyl | *refrigerator* | /ɕøːk/ | INI | 0 |
| chips | *crisps* | /ɕɪps/ | INI + FIN | 0 |
| ambulans | *ambulance* | /ambɵˈlans/ | FIN | 1 |
| glasögon | *eye glasses* | /ɡl^2^ɑːsˌøːɡɔn/ | MED | 0 |
| sax | *scissors* | /saks/ | INI + FIN | 0 |
| hals | *neck* | /hals/ | INI + FIN | 1 |
| tuschpenna | *marker pen* | /t^2^ɵʃˌpɛna/ | MED | 1 |
| byxor | *pants* | /b^2^ʏksʊr/ | MED | 1 |
| apelsin | *orange* | /apɛlˈsiːn/ | MED | 0 |
| svans | *tail* | /svans/ | INI + FIN | 1 |
| sjöhäst | *sea hoarse* | /^2^ɧøːˌhɛst/ | INI + MED | 0 |
| mustasch | *moustache* | /mɵˈstɑːʃ/ | FIN | 1 |
| tandborste | *tooth brush* | /^2^tandˌbɔʂʈə/ | MED | 0 |
| potatis | *potato* | /pʊˈtɑːtɪs/ | FIN | 0 |
| kök | *kitchen* | /ɕøːk/ | INI | 1 |
| sjuk | *ill* | /ɧʉːk/ | INI | 1 |
| sked | *spoon* | /ɧeːd/ | INI | 0 |
| snor | *snot* | /snuːr/ | INI | 0 |
| köttfärssås | *meat sauce* | /^2^ɕøtfæˌʂoːs/ | MED + FIN | 0 |
| sand | *sand* | /sand/ | INI | 1 |
| explosion | *explosion* | /ɛksplɔˈɧuːn/ | MED | 1 |
| choklad | *chocolate* | /ɧʊˈklɑːd/ | INI | 1 |
| spindel | *spider* | /^2^spɪndɛl/ | INI | 0 |

*Table S2.* Word probes (*n* = 24) selected for participants with intervention targeting velar fronting. (Note that a single word can contain multiple target consonants, i.e. velar consonants.)

| **Word** | ***English translation*** | **Target form** | **Target sound word position(s)** | **Trained (0=no, 1=yes)** |
| --- | --- | --- | --- | --- |
| klocka | *watch* | /klɔka/ | INI + MED | 0 |
| igelkott | *hedgehog* | /iːɡɛlˌkɔt/ | MED + MED | 1 |
| cykel | *bike* | /ˈsʏkɛl/ | MED | 1 |
| ballong | *balloon* | /baˈlɔŋ/ | FIN | 0 |
| explosion | *explosion* | /ɛksplɔˈɧuːn/ | MED | 1 |
| såg | *saw* | /so:ɡ/ | FIN | 0 |
| sax | *scissors* | /saks/ | MED | 0 |
| balkong | *balcony* | /balˈkɔŋ/ | MED + FIN | 1 |
| hink | *bucket* | /hɪŋk/ | MED + FIN | 0 |
| jordgubbe | *strawberry* | /juːɖˌɡɵbə/ | MED | 0 |
| katt | *cat* | /kat/ | INI | 1 |
| gunga | *swing* | /ɡɵŋa/ | INI + MED | 1 |
| ägg | *egg* | /ɛɡ/ | FIN | 1 |
| barnvagn | *stroller* | /bɑːɳˌvaŋn/ | MED | 0 |
| glass | *ice cream* | /ɡlas/ | INI | 1 |
| glasögon | *eye glasses* | /ɡlɑːsˌøːɡɔn/ | INI + MED | 0 |
| byxor | *pants* | /bʏksʊr/ | MED | 1 |
| glödlampa | *light bulb* | /ɡløːdˌlampa/ | INI | 1 |
| biosalong | *movie theatre* | /biːʊsaˌlɔŋ/ | FIN | 1 |
| ring | *ring* | /rɪŋ/ | FIN | 0 |
| gräs | *grass* | /ɡrɛːs/ | INI | 0 |
| tåg | *train* | /to:ɡ/ | FIN | 0 |
| kalsonger | *underpants* | /kalˈsɔŋər/ | MED | 0 |
| bok | *book* | /buːk/ | FIN | 1 |

*Table S3.* Words (*n =* 86) included as targets in the speech training game, for intervention targeting stopping. (Sorted in alphabetical order.)

| **Target (Sw)** | ***English*** | **Target (Sw)** | ***English*** | **Target (Sw)** | ***English*** |
| --- | --- | --- | --- | --- | --- |
| affisch | *poster* | kalas | *party* | sjösjuk | *sea sick* |
| ambulans | *ambulance* | kanske | *maybe* | sjöstjärna | *starfish* |
| ananas | *pineapple* | kasta | *throw* | skateboard | *skateboard* |
| andas | *breathe* | kilo | *kilogram* | skjorta | *shirt* |
| aprikos | *apricot* | kollision | *collision* | skogsdunge | *grove* |
| avgas | *exhaust* | kompis | *friend* | skolklass | *school class* |
| balansgång | *balancing* | kusin | *cousin* | skridskor | *skates* |
| bekämpa | *fight* | kändis | *celebrity* | skriva | *write* |
| biosalong | *movie theater* | kärna | *core* | slag | *blow* |
| buss | *bus* | köpa | *buy* | socker | *sugar* |
| byxor | *trousers* | kött | *meat* | soffa | *sofa* |
| bäst | *best* | leksaksbil | *toy car* | springa | *run* |
| choklad | *chocolate* | ljust | *bright* | språk | *language* |
| dusch | *shower* | läskig | *scary* | stark | *strong* |
| energi | *energy* | läxa | *homework* | station | *station* |
| erkänna | *confess* | motion | *exercise* | stjärna | *star* |
| explosion | *explosion* | mus | *mouse* | störst | *biggest* |
| fisk | *fish* | musik | *music* | svans | *tail* |
| fotbollslag | *soccer team* | mustasch | *mustache* | svart | *black* |
| frisk | *healthy* | okänd | *unknown* | sång | *song* |
| först | *first* | orange | *orange* | säga | *say* |
| garage | *garage* | prins | *prince* | tjej | *girl* |
| gelé | *jelly* | ryggsäck | *backpack* | trasig | *broken* |
| glass | *icecream* | sand | *sand* | tuschpenna | *marker pen* |
| godis | *candy* | sju | *seven* | usch | *yuk* |
| gris | *pig* | sjuk | *sick* | version | *version* |
| hals | *neck* | sjukhus | *hospital* | väska | *bag* |
| hus | *house* | sjunga | *sing* | ögonfrans | *eyelash* |
| information | *information* | sjö | *lake* |  |  |

*Table S4.* Words (*n =* 93) included as targets in the speech training game, for intervention targeting velar fronting.

| **Target (Sw)** | ***English*** | **Target (Sw)** | ***English*** | **Target (Sw)** | ***English*** |
| --- | --- | --- | --- | --- | --- |
| aprikos | *apricot* | gunga | *swing* | läskig | *scary* |
| balansgång | *balancing* | gård | *courtyard* | läxa | *homework* |
| balkong | *balcony* | hage | *pasture* | mjuka | *soft* |
| biosalong | *movie theater* | hagel | *hail* | musik | *music* |
| bok | *book* | helikopter | *helicopter* | målning | *painting* |
| bygga | *build* | hungrig | *hungry* | pengar | *money* |
| choklad | *chocolate* | igelkott | *hedgehog* | ryggsäck | *backpack* |
| cykel | *bike* | jaguar | *jaguar* | sjuk | *sick* |
| dag | *day* | jättelång | *very long* | sjukhus | *hospital* |
| deg | *dough* | kaka | *cookie* | sjunga | *sing* |
| drag | *move* | kalas | *party* | sjösjuk | *seasick* |
| dryck | *beverage* | kanin | *rabbit* | skateboard | *skateboard* |
| finger | *finger* | kanske | *maybe* | skogsdunge | *grove* |
| fisk | *fish* | kasta | *throw* | skolklass | *school class* |
| fotbollslag | *soccer team* | katt | *cat* | skridskor | *skates* |
| frisk | *healthy* | kattunge | *kitten* | skriva | *write* |
| företag | *company* | klack | *heel* | socker | *sugar* |
| förslag | *suggestion* | kollision | *collision* | springa | *run* |
| galopp | *gallop* | kompis | *friend* | språk | *language* |
| gammal | *old* | kort | *short* | stark | *strong* |
| gapa | *gape* | krage | *collar* | sång | *song* |
| garage | *garage* | kropp | *body* | tack | *thanks* |
| glass | *icecream* | kung | *king* | taggig | *prickly* |
| glödlampa | *bulb* | kusin | *cousin* | trång | *narrow* |
| godis | *candy* | kök | *kitchen* | ugn | *oven* |
| granne | *neighbour* | lag | *team* | vagga | *cradle* |
| gris | *pig* | leka | *play* | väg | *road* |
| groda | *frog* | leksaksbil | *toy car* | väska | *bag* |
| gråta | *cry* | lok | *locomotive* | ägg | *egg* |
| gröt | *porridge* | lugnt | *calm* | äng | *field* |
| gul | *yellow* | lyftkran | *crane* | ögonfrans | *eyelash* |

*Appendix S5.* User experience questionnaire (in its original Swedish form, and its English translation), distributed to the child users after the intervention, for them to fill out with the assistance of their caregivers.

| *Swedish (original)* | *English (translation)* |
| --- | --- |
| **Användarupplevelser**  *Vi vill gärna veta vad du tyckte om spelet, och är tacksam om du kan dela med dig av dina synpunkter.*  *Tack!*  [Svarsalternativ: Inte alls/Lite/Ganska mycket/Mycket]   1. Jag gillade spelet 2. Spelet var lätt 3. Jag gillade att höra min egen röst i spelet 4. Jag gillade att få stjärnor 5. Efter att ha tränat med spelet har jag blivit bättre på att säga de ljud som är svåra för mig 6. Jag vill träna mer på de ljud som är svåra för mig 7. Jag vill spela spelet igen   [Fritext]   - Hur skulle spelet kunna bli bättre? - Vill du säga något mer om spelet? | **User experiences**  *We would like to know what you thought about the game, and would be greatful if you could share your experiences.*  *Thank you!*  [Response alternatives: Not at all/A little/Pretty much/A lot]   1. I liked the game 2. The game was easy 3. I liked hearing my own voice in the game 4. I liked receiving stars 5. After having practiced with the game, I’ve become better at saying the sounds that are difficult for me 6. I’d like to practice more on saying the sounds that are difficult for me 7. I’d like to play the game again   [Fritext]   - What would make the game better? - Is there anything more you’d like to say about the game? |

*Table S6.* Overview over the distribution of star ratings provided as feedback to the production of target words, for the four participants.

|  | **n (%) ratings per rating category** | | | | |  |
| --- | --- | --- | --- | --- | --- | --- |
|  | **1** | **2** | **3** | **4** | **5** | **Total n ratings** |
| **Child** |  |  |  |  |  |  |
| Adam | 21 (58%) | 9 (25%) | N/A | 5 (14%) | 1 (3%) | 36 |
| Benjamin | 65 (59%) | 11 (10%) | N/A | 18 (16%) | 16 (15%) | 110 |
| Carl | 68 (57%) | 16 (13%) | 1 (1%) | 18 (15%) | 17 (14%) | 120 |
| Doris | 38 (53%) | 8 (11%) | N/A | 10 (14%) | 16 (22%) | 72 |
